# Rethinking team science metrics through Collaborative Guideposts

**DOI:** 10.3389/fpsyg.2026.1717847

**Published:** 2026-04-10

**Authors:** Kristen J. McQuerry, Kelsey Karnik, Megan E. Hall, Caitline Phan, Yana Feygin, Emily Slade

**Affiliations:** Department of Biostatistics, University of Kentucky, Lexington, KY, United States

**Keywords:** collaboration metrics, interdisciplinary teams, research evaluation, team science, translational impact

## Abstract

**Introduction:**

The increasing reliance on interdisciplinary and transdisciplinary teams has reshaped how science is conducted, yet evaluating the success of such teams remains a persistent challenge. Traditional metrics, such as publication counts, impact factors, and citation indices, fail to capture the full scope of team contributions, particularly in contexts where success may be defined by community engagement, mentorship, or long-term collaboration.

**Methods:**

In this Perspective, we introduce Collaborative Guideposts, a framework designed to align success metrics with a team’s organizing priorities. We illustrate this approach through two contrasting team models from a biostatistical collaboration center: Team A, a time-bound, community-focused group emphasizing rapid deliverables and public health partnerships, and Team B, a mentorship-driven, development-focused group prioritizing capacity-building and long-term scholarly growth.

**Results:**

From these cases, we derive four Collaborative Guideposts: Time Perspective, Unit of Assessment, Desired Outcomes, and Sustainability. These Guideposts provide a structured yet flexible way to capture team success.

**Discussion:**

By grounding existing evaluation in Guideposts that reflect team-specific goals, this framework supports more accurate and meaningful assessments of collaborative impact. Our contribution responds to calls for validated models of team science by offering a pragmatic, adaptable framework that bridges theory and practice, enabling both immediate evaluation and long-term sustainability of scientific collaborations.

## Introduction

Collaborative teams come in many shapes and sizes, each with distinct structures, priorities, and pathways to impact. Teams are increasingly the strategy of choice when organizations confront tasks too complex for individuals, requiring high interdependence, rapid decision-making, and shared responsibility (Salas et al., 2008; Salas et al., 2018). The growing importance of such collaborations, from both an intellectual and funding standpoint, has fundamentally shifted how science is conducted and how new scholars are prepared for careers in science (Begg et al., 2014). Investigators increasingly recognize that solving complex problems requires working across disciplinary and institutional boundaries, yet forming a successful team is rarely a matter of chance (Waite et al., 2023). National reports have similarly emphasized that addressing complex societal and scientific challenges increasingly requires convergence across disciplines, sectors, and methodological approaches, underscoring the need for intentional team structures that support integration and coordination (National Academies of Sciences, Engineering, and Medicine, 2014). Teams take time to merge, build trust, and function at a high level, and early stages may initially produce modest returns as foundational relationships and systems are established.

A body of work has identified core elements that reinforce productive team science, including intentional participant selection, shared goals, accountability, equitable credit, clear communication, leadership and mentoring, conflict resolution, and sustained advancement (Waite et al., 2023). These elements require deliberate implementation, often from the outset, through mechanisms like team agreements, data management systems, and structured mentorship programs (Bolduc et al., 2023). Beyond these structural elements, recent work emphasizes the importance of interactional expertise and relational processes, such as equitable turn-taking and inclusive participation, as predictors of whether collaborations succeed or falter (Love et al., 2022).

Despite the well-documented benefits of team science, assessing its success remains a persistent challenge. Traditional metrics, such as publication counts, impact factors, citations, and h-indices, are limited in their ability to capture creativity, translational impact, and team development (Duda et al., 2023). Comprehensive reviews highlight that most existing studies rely heavily on bibliometric indicators and pre-existing data, leaving a gap in process-oriented and longitudinal evaluations (Hall et al., 2018). Scholars increasingly argue that a multidimensional approach to assessing scholarly impact is required (Moed and Halevi, 2015). The reliance on publication-centric metrics as the primary metric of success can undervalue slower-developing, highly collaborative work that may yield significant societal benefit but accrue traditional markers at a delayed pace (Dirnagl et al., 2022; Pasterkamp et al., 2016). This misalignment is especially critical for early-career scientists, who must balance the internal motivation to pursue meaningful research with the external pressures of career advancement. Competency-based frameworks for clinical and translational researchers offer one alternative, but validated assessments remain limited and inconsistently applied (Ianni et al., 2021). Broader conceptual models, such as those developed in the Clinical and Translational Science Award program, underscore the importance of evaluating both external indicators (e.g., funding, promotion) and internal factors (e.g., satisfaction, mentoring, collaboration) to capture the full spectrum of career outcomes (Lee et al., 2012).

Scientific collaboration is not only an intellectual endeavor, but also a social process, dependent on shared research questions, collective planning, and coordinated execution (Graesser et al., 2018). Science facilitation, encompassing both intellectual guidance and logistical coordination, plays a pivotal role in ensuring that resources, time, and effort are effectively managed (Cravens et al., 2022). Understanding how facilitation and leadership roles interact with a team’s core priorities is essential for designing and sustaining effective collaborations.

The broader science-of-team-science literature has long emphasized the need for validated conceptual models and rigorous evaluation frameworks to capture the diverse ways teams achieve success (Stokols et al., 2008). Because team type, context, and developmental stage vary widely, success metrics must align closely with team goals (Mâsse et al., 2008). While evidence-based models and measurement tools exist (Wildman and Bedwell, 2013; Mâsse et al., 2008), their adoption, adaptation, and validation across diverse research settings remain limited.

Recent work further emphasizes that progress in research assessment will not necessarily come from inventing new metrics, but from existing, validated measures within team-based frameworks. Bennett et al. (2023) argue for systemic reforms of Team Infrastructure Roles whose contributions sustain scientific progress but are rarely rewarded under traditional evaluation models. At the team level, Hernandez et al. (2021) show that self-assessment can improve communication, well-being, and inclusion, offering a practical approach to team development. At the institutional level, McKiernan et al. (2019) and McKiernan et al. (2024) document how embedded the use of journal impact factors in promotion and tenure reviews can be replaced by value-based approaches that prioritize equity, openness, and mentorship. Together, these studies reinforce that success frameworks should draw upon validated instruments, but be restructured to reflect each team’s purpose, timeline, and social dynamics.

In this Perspective, we build on this principle by proposing Collaborative Guideposts. These Guideposts are deliberate markers established at a team’s initiation that align success metrics with its organizing priorities. Rather than creating new tools for evaluation, the Guideposts provide a practical scaffold for selecting existing and established measures to ensure that what teams track truly reflects what makes them successful. We examine their application using two distinct team models from our institution’s biostatistical collaboration center and explore how “success” can be defined and measured in contexts with noticeably different team structure and goals. The team models described are drawn from the authors’ direct experiences within a biostatistical collaboration center and are intended to serve as illustrative examples rather than exhaustive case studies. By grounding existing, established evaluation criteria in these Guideposts, teams can more effectively capture their intended impact, balance short-term productivity with long-term investment, and sustain engagement over time. Our case examples illustrate how Collaborative Guideposts can serve as practical contributions to the development and validation of team science models.

## Team models

The following sections introduce two team models from our institution in which the authors of this paper were directly involved, each with distinct structures and priorities. These examples are presented as illustrative case models to provide context for how teams operate in practice before turning to the concept of Collaborative Guideposts.

### Team A

Team A represents a highly organized, time-bound research group formed to build community partnerships and produce deliverables within a one-year period. This team received internal funding with the objective of creating a public health action team to facilitate tailored responses to emerging health needs at a community hospital. A single integrated team was formed, with outcomes collectively defined and executed. Rather than pursuing multiple projects simultaneously, the team worked collectively to complete each initiative one by one. The team was intentionally composed of partners across domains and career levels, including research faculty, research staff, clinical experts, community hospital staff, and community partners. Each member contributed distinct skills and expertise aligned with the team’s overarching goals.

Community engagement meetings were structured around hard deadlines for analyses, ensuring timely progress. Each meeting had a specific area of focus to streamline processes and maximize relevance to community partners. During these meetings, members reviewed and interpreted findings and advised on next analytical steps for intervention planning. In the first community engagement meeting, the team reviewed findings to identify needs for tailored community or health system interventions to increase colorectal cancer screening rates and co-design intervention strategies using established design thinking methods. This structured approach to community engagement proved highly successful, resulting in multiple presentations and a manuscript that described and disseminated the community engagement model (McGladrey et al., 2025).

Overall, Team A emphasized centralized coordination, shared ownership of goals, strong leadership, yielding timely scholarly outputs and setting the stage for future collaboration with the community hospital beyond the original funding period. Following the initial project, collaboration with the hospital continued, though with new team members and different objectives.

For similar time-bound research teams aligned under a common, clearly defined research objective, potential success metrics may be selected from existing, established indicators and categorized according to the relevant Collaborative Guideposts. Examples include:

- Productivity: number/duration of deliverables completed vs. planned.
- Engagement: number of partner meetings; implementation of recommendations.
- Sustainability: subsequent funding; new partnerships emerging.

The time-bound approach limits opportunities to pursue long-term initiatives within the original team structure, particularly those tailored to individual members or evolving contexts. For instance, there were multiple research questions that the team wanted to address but were only able to complete one project within the time constraint.

Time-bound teams must be realistic about their outcome expectations. For a team focused primarily on community engagement, it may be tempting to define success as immediate community-level impact; however, such outcomes typically occur beyond the scope of a one-year funding period. Instead, success should be defined through process-based or short-term metrics. Actionable analyses, partner engagement, and dissemination are examples of products that can set the stage for longer term impacts to be evaluated by subsequent teams.

Team A illustrates a highly structured, time-sensitive model designed to achieve rapid and focused community impact. In the next section, we turn to a contrasting team model that reflects a different set of priorities and collaborative dynamics. Together, these two examples provide insights into the diverse ways team science can operationalize Collaborative Guideposts to meet distinct goals.

### Team B

Team B demonstrates a contrasting approach. This second team emphasizes mentorship, flexibility, and long-term development, revealing how different team structures and priorities shape collaborative processes and outcomes.

Team B represents a mentorship-driven research group, established by a senior faculty member to support early-career investigators across diverse disciplines. This team comprises roughly 26 members, each pursuing an independent project within a shared chronic disease focus area. While the group meets regularly as a whole, the structure encourages each investigator to develop a distinct area of expertise within the broader research domain.

The senior faculty member leading the team emphasizes quality over speed, often opting to forego conference abstract submission deadlines if an analysis would need to be rushed, preferring to invest the necessary time to ensure robust, novel, and thoughtful research. In contrast to Team A’s linear, milestone-driven structure, Team B members advance through projects at different paces, each pursuing individual goals that collectively support the broader team mission. Each team member is responsible for reporting their project timelines to ensure progress is maintained without compromising quality.

With its flexible timeline and broader project scope, this team model prioritizes long-term development, sustained collaboration, and the mentorship of early-career faculty, even if traditional academic productivity metrics accrue more gradually. The Collaborative Guideposts framework provides a structure for teams like Team B to select from established, existing measures that best represent their developmental priorities rather than creating new evaluation tools. For the mentorship-driven team, potential success metrics could include:

- Capacity Building: number of early-career faculty mentored; evidence of mentee growth or independence (first-authored publications, new collaborations).
- Productivity: manuscripts started or submitted, grant submissions and awards.
- Collaboration: number of cross-disciplinary partnerships formed; new ideas generated.
- Sustainability: career progression (promotion, tenure, retention) and continuation of mentoring relationships.

Collaborative Guideposts also encourage teams to prioritize metrics that match leadership goals. For instance, if the senior PI’s priority is developing new research ideas, pilot-funding applications or secondary-data publications may be key indicators. If expanding existing research programs is the aim, larger-scale submissions (e.g., R21s, R01s) may serve as success metrics. From an early career-faculty perspective, frequent mentorship meetings or explicit career-trajectory discussions may hold more value.

The potential limitation of this model is that these longer term metrics may not align with departmental and institutional review timelines, which often prioritize rapid, publication-based outputs. Embedding Collaborative Guideposts during team formation allows for clearer documentation of developmental progress that may not be captured by conventional measures. This helps to ensure that achievements such as mentoring quality, network growth, and research independence, are visible and valued.

## Team success metrics

These contrasting approaches of Teams A and B raise an important consideration: how do we define and measure success on teams with very different goals? Identifying each team’s Collaborative Guidepost is critical for selecting metrics that meaningfully capture its intended impact.

For Team A, time was the critical factor; thus, organization, clear leadership, and cohesive teamwork were essential to produce outputs within the funding period. For Team B, mentorship was the central priority. Success metrics in this model were more individualized, reflecting each early-career investigator’s specific goals and developmental needs. In such a context, traditional measures of productivity may not fully capture success, which may instead be reflected in the growth, independence, and scholarly maturation.

We propose four Collaborative Guideposts for aligning success metrics with team priorities:

1) Time Perspective: short-term vs. long-term goals.
2) Unit of Assessment: individual vs. team-based metrics.
3) Desired Outcomes: scholarly products, innovation, translation to practice, community impact.
4) Sustainability: ongoing collaboration, funding, and capacity-building.

These Guideposts offer teams a flexible framework to track success ensuring alignment with their unique goals and environments. The Collaborative Guideposts do not replace validated evaluation tools; rather, they help teams organize and apply existing measures in ways that reflect their specific context and purpose.

**Figure 1:**
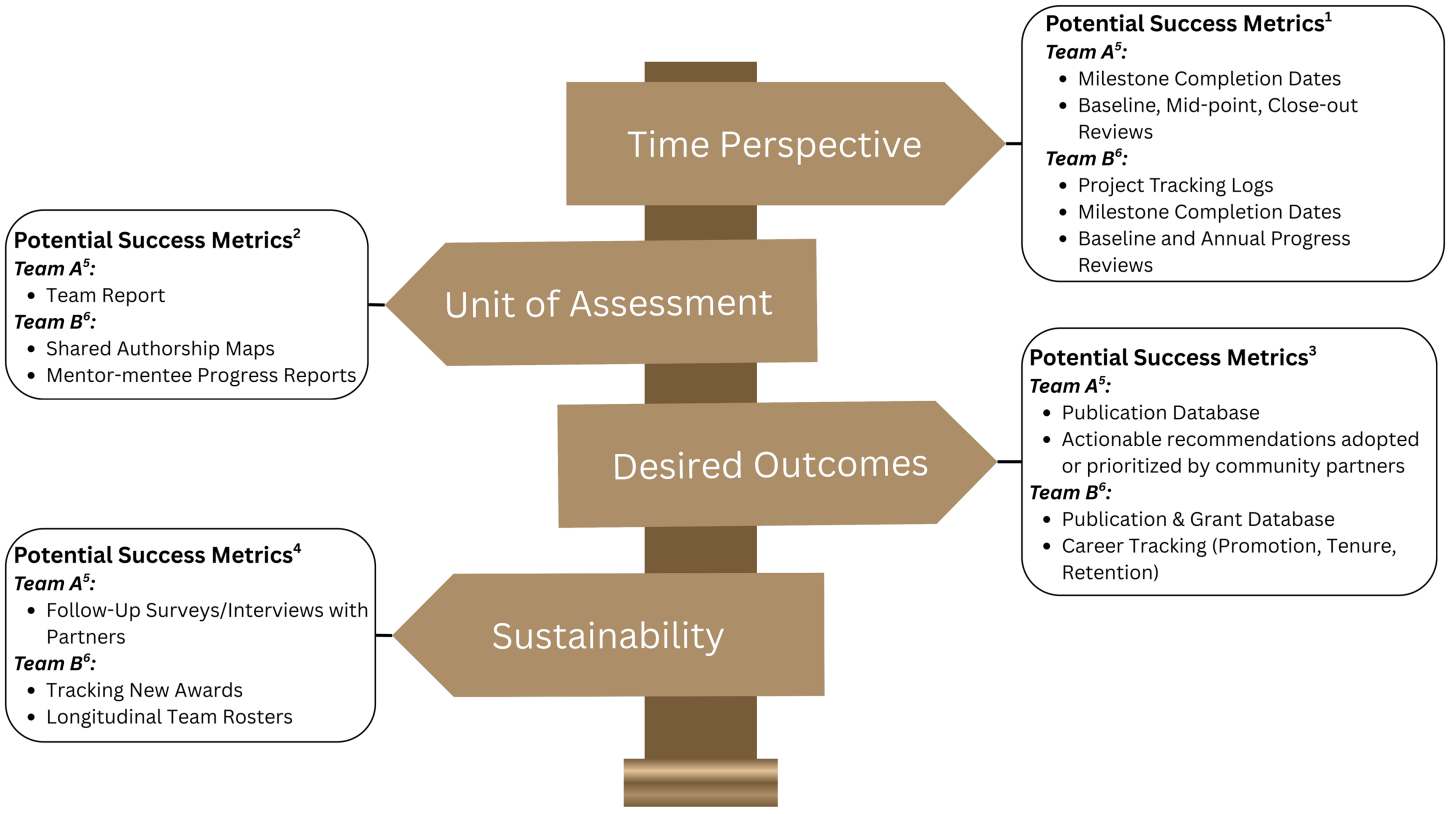
Collaborative guideposts with potential outcome measures 1) Timing and developmental stage are critical considerations for team evaluation (Stokols et al., 2008; Mâsse et al., 2008). 2) Multilevel analyses that capture both individual and team dynamics have been recommended for team science evaluations (Wildman and Bedwell, 2013). 3) Traditional bibliometric outcomes should be complemented by developmental and translational impact measures (Duda et al., 2023; Dirnagl et al., 2022). 4) Sustainability and long-term impact through partnerships, mentoring, and continued funding have been emphasized as central to effective team science (Bolduc et al., 2023; Cravens et al., 2022). 5) Team A is time-bound and community-focused. 6) Team B is mentorship-driven and development-focused.

To illustrate how Collaborative Guideposts can be applied, Table 1 compares the Collaborative Guideposts for two contrasting team models. Figure 1 shows how existing, established success metrics can be organized within the Collaborative Guideposts framework. By mapping measurable outcomes to Time Perspective, Unit of Assessment, Desired Outcomes, and Sustainability Guideposts, teams can align evaluation criteria with their specific structure and goals.

**Table 1:** Collaborative guideposts for two team models.

Collaborative guidepost	Team A (time-bound, community-focused)	Team B (mentorship-driven, development-focused)
Time perspective	Short-term deliverables completed within 1-year funding cycle (e.g., analyses, community meeting presentations, manuscript)	Long-term (ongoing) development of independent projects by early-career faculty
Unit of assessment	Team-level ownership of outcomes; single integrated project structure	Individual-level progress within shared research theme; team-level mentoring and support
Desired outcomes	Number of deliverables (e.g., community meeting presentations, manuscript, implemented recommendations by partners)	Scholarly products (e.g., pilot grant submissions, publications); career progression of mentees
Sustainability	New partnerships, subsequent funding, continued engagement with community hospital and partners	Continued collaboration, renewal or procurement of funding (e.g., KL2, R01), sustained mentoring chains

Our proposed Guideposts contribute to the broader aim of advancing validated models of team science by offering a flexible yet structured framework for aligning established success metrics with the appropriate priority. Each team can determine the relative importance of each Guidepost and identify which existing measures best capture their progression and success.

While the Collaborative Guideposts are designed to evaluate team-level performance, individual members also benefit from a team’s success. Effective team functioning such as shared leadership, mentoring, and sustainability can be captured through these Guideposts. This, in turn, strengthens individual visibility, enhances scholarly productivity, and provides tangible evidence of contribution in promotion and tenure evaluations. Collaborative Guideposts help teams demonstrate collective impact while simultaneously supporting individual advancement within institutional frameworks.

## Discussion

This Perspective contributes a practical framework, Collaborative Guideposts, for aligning success metrics with the diverse goals of research teams, offering both conceptual and applied examples to guide evaluation in real-world settings.

Team membership does not need to look a certain way for Guideposts to be useful. Teams of all sizes, disciplines, and compositions can contribute to defining their Collaborative Guidepost so that the outcomes are meaningful and relevant. New instruments for measuring success do not need to be created; rather, existing and established metrics can be integrated within the Collaborative Guidepost structure to capture what makes each team successful.

Although these Guideposts provide the flexibility to track team success, there are several considerations in their practical application. First, operationalization requires intentionality. A team initiation document may be useful to record agreed-upon Guideposts and success metrics at the time of team creation. Determining who authors and maintains this document is crucial. If drafted solely by leadership, it may reflect hierarchical priorities rather than shared values. Ideally, the process of defining Guideposts should be co-created, ensuring early-career investigators, staff, and community partners all have input into what counts as meaningful success. This process also highlights disciplinary variation: what constitutes a key deliverable in biomedical sciences may differ markedly from social sciences, community-engaged research, or other fields.

Second, the timing of assessment is critical. Teams differ in their cadence of activity: a time-bound project may require baseline, mid-point, and closeout evaluations, whereas a mentorship-driven team might benefit from yearly and longitudinal reviews. Relying solely on end-point evaluation risks missing formative insights that could strengthen team functioning midstream. Here, Collaborative Guideposts can serve as “mid-course correction” tools, much like continuous quality improvement processes in healthcare. Real-time or periodic feedback can allow adjustments before issues derail progress.

Third, measurement tools warrant careful consideration. While validated surveys of team processes exist (Wildman and Bedwell, 2013), they are not yet widely adopted across disciplines. Mâsse et al., 2008 emphasized the need for robust measures of transdisciplinary integration, while Stokols et al. (2008) called for multilevel approaches that capture outcomes at the individual, team, and institutional levels. By embedding validated surveys, bibliometric analyses, and career-tracking systems into team evaluations, Collaborative Guideposts can be linked to empirically grounded indicators rather than *ad hoc* measures.

Another challenge is institutional alignment. While Collaborative Guideposts may capture a team’s true contributions, many of these metrics (e.g., mentoring quality, community partnerships, shared leadership) are undervalued in traditional promotion and tenure reviews, which typically prioritize individual publications and grant dollars. Without intentional institutional alignment, teams risk a misfit between internal definitions of success and external evaluation systems. Departments and institutions could address this by piloting Guidepost-based metrics in annual reviews or creating supplemental reports that document team leadership, mentoring impact, and collaborative outputs. Although academic advancement is assessed at the individual level, the success of a team can directly contribute to and verify an individual’s professional growth, visibility, and impact when guided by the Collaborative Guideposts.

The risks and benefits of adopting or neglecting Guideposts also deserve attention. Without clear Guideposts, teams may default to narrow measures of success, undervaluing contributions that matter most to team members and stakeholders. This can lead to frustration, inequitable recognition, and even premature team dissolution. At the same time, adopting Guideposts is not without risk. Explicit evaluation frameworks can introduce additional complexity and time demands, and if implemented rigidly, may divert attention away from the scientific or collaborative work itself. There is also a risk that Guideposts defined in a top-down manner may reinforce hierarchical priorities rather than reflect shared team values. As discussed earlier, these risks underscore the importance of proportionality and co-creation. When used as lightweight, intentionally designed tools rather than exhaustive reporting requirements, Collaborative Guideposts can support reflection and alignment without overwhelming teams or constraining their work.

Finally, these insights contribute to advancing the broader science-of-team-science field. Our proposed Collaborative Guidepost framework responds directly to calls for validated conceptual models (Stokols et al., 2008; Mâsse et al., 2008) by offering a pragmatic approach that bridges theory and practice. Collaborative Guideposts are not intended to replace existing models but rather to serve as applied scaffolds that are practical markers, connecting abstract constructs to daily team management. Future research should test whether teams that set Guideposts early are more likely to sustain collaborations, secure continued funding, achieve translational impact, and have higher member satisfaction and engagement throughout the team’s lifespan.

Collaborative Guideposts provide a structured yet flexible way to not only align existing evaluation criteria with team-specific contexts, but also to initiate team-level discussions of outcome goal. Teams can determine which Guidepost and outcomes are of most importance and adapt their framework so that success is measured in ways that reflect both scientific progress and collaborative quality. By illustrating their application through two contrasting team models, we highlight both the promise and complexity of operationalizing success in team science. Our approach underscores the importance of validated, adaptable frameworks to ensure that teams are evaluated not only for what they produce, but for how they collaborate, mentor, and sustain long-term scientific progress.

## Data Availability

The original contributions presented in the study are included in the article/supplementary material, further inquiries can be directed to the corresponding author.
